# Dendritic Arborization Patterns of Small Juxtaglomerular Cell Subtypes within the Rodent Olfactory Bulb

**DOI:** 10.3389/fnana.2016.00127

**Published:** 2017-01-20

**Authors:** Wolfgang G. Bywalez, Tiffany Ona-Jodar, Michael Lukas, Jovica Ninkovic, Veronica Egger

**Affiliations:** ^1^Systems Neurobiology, Department II of Biology, Ludwig-Maximilians-Universität MünchenMunich, Germany; ^2^Neurophysiology, Institute of Zoology, Universität RegensburgRegensburg, Germany; ^3^Institute for Stem Cell Research, Helmholtz Zentrum München, German Research Center for Environmental Health (GmbH)Munich, Germany; ^4^Institute of Physiological Genomics, Ludwig-Maximilians-Universität MünchenMunich, Germany; ^5^Cluster for Systems Neurology and BioMedical Center, Ludwig-Maximilians-Universität MünchenMunich, Germany; ^6^Regensburg Center of Neuroscience, Universität RegensburgRegensburg, Germany

**Keywords:** juxtaglomerular cells, dopaminergic neurons, subcellular compartments, dendritic arborization analysis, calcium imaging, dendrites, two-photon imaging, morphological reconstruction

## Abstract

Within the glomerular layer of the rodent olfactory bulb, numerous subtypes of local interneurons contribute to early processing of incoming sensory information. Here we have investigated dopaminergic and other small local juxtaglomerular cells in rats and mice and characterized their dendritic arborization pattern with respect to individual glomeruli by fluorescent labeling via patching and reconstruction of dendrites and glomerular contours from two-photon imaging data. Dopaminergic neurons were identified in a transgenic mouse line where the expression of dopamine transporter (DAT) was labeled with GFP. Among the DAT+ cells we found a small short-axon cell (SAC) subtype featuring hitherto undescribed dendritic specializations. These densely ramifying structures clasped mostly around somata of other juxtaglomerular neurons, which were also small, non-dopaminergic and to a large extent non-GABAergic. Clasping SACs were observed also in wild-type mice and juvenile rats. In DAT+ SAC dendrites, single backpropagating action potentials evoked robust calcium entry throughout both clasping and non-clasping compartments. Besides clasping SACs, most other small neurons either corresponded to the classical periglomerular cell type (PGCs), which was never DAT+, or were undersized cells with a small dendritic tree and low excitability. Aside from the presence of clasps in SAC dendrites, many descriptors of dendritic morphology such as the number of dendrites and the extent of branching were not significantly different between clasping SACs and PGCs. However, a detailed morphometric analysis in relation to glomerular contours revealed that the dendrites of clasping SACs arborized mostly in the juxtaglomerular space and never entered more than one glomerulus (if at all), whereas most PGC dendrites were restricted to their parent glomerulus, similar to the apical tufts of mitral cells. These complementary arborization patterns might underlie a highly complementary functional connectivity. The morphometric approach may serve to differentiate also other subtypes of juxtaglomerular neurons, help to identify putative synaptic partners and thus to establish a more refined picture of glomerular network interactions during odor sensing.

## Introduction

The mammalian olfactory bulb (OB) provides a two-stage network formed by groups of local interneurons, with the first stage located within the glomerular layer, where numerous subtypes of glomerular interneurons contribute to early processing of incoming sensory information (Wachowiak and Shipley, 2006).

The most numerous juxtaglomerular cells (JGCs) are GABAergic periglomerular cells (PGCs, Parrish-Aungst et al., 2007). These small interneurons usually project with their dendrites into one or at most two glomeruli (Nagayama et al., 2014). Some PGCs appear to bear a locally projecting axon whereas other subtypes are axonless (Pinching and Powell, 1971c; Kosaka and Kosaka, 2011). They exert inhibition onto olfactory nerve (ON) terminals and many cell types in the glomerular layer which include mitral and tufted cells (MTCs), external tufted cells (ETCs), other PGCs and short axon cells (SACs) (Pinching and Powell, 1971a,c; Kosaka et al., 1998).

Another large subgroup of juxtaglomerular neurons, the dopaminergic/GABAergic JGCs, are nowadays mostly considered as SACs instead of the traditional classification as PGCs or even ETCs, since they exhibit multiglomerular innervation (Kiyokage et al., 2010). They play important roles in mammalian olfactory function, including stimulus gating, gain control, and pacemaker activity (Pignatelli et al., 2005; Liu et al., 2013; Banerjee et al., 2015). In mice, all JGCs expressing tyrosine hydroxylase (TH+), the most established marker for dopaminergic cells in the OB, co-express GABA (Kosaka and Kosaka, 2007; Parrish-Aungst et al., 2007), which also holds true for the majority of TH+ cells in the rat (Gall et al., 1987; Kosaka et al., 1995). Similar to PGCs, their postsynaptic targets include MTCs, ETCs and other glomerular interneurons, and they can also mediate presynaptic inhibition of ON terminals (Cave and Baker, 2009).

While by now the term “short axon cell” is known to be a misnomer, the neurite arborization patterns and therewith the proper classification of dopaminergic JGCs are still under debate: Originally, glomerular layer SACs were observed to ramify mostly or exclusively in the juxtaglomerular space (e.g., Pinching and Powell, 1971a). Recently, Kiyokage et al. (2010) reported an extended arborization of small dopaminergic SACs within several to dozens of glomeruli, without differentiating between axons and dendrites. Based on this glomerular, apparently dendritic innervation other authors have proposed to label the dopaminergic neurons as conventional PGCs (Kosaka and Kosaka, 2011). Here we attempt to resolve some of the controversy by providing a detailed description of dendritic arborization patterns of small glomerular interneurons, both dopaminergic and non-dopaminergic.

Our study describes small dopamine transporter expressing (DAT+) neurons and other small JGCs from acute mouse or rat brain slices. DAT+ cells were identified in a transgenic mouse line by their conditional tamoxifen-induced expression of GFP (Rieker et al., 2011), whereas other neuronal morphologies were obtained by random patching of JGCs with small somata. Individual neurons were filled with fluorescent dye and their dendrites were reconstructed from two-photon laser scans along with the contours of contacted or closely surrounded glomeruli that could be detected in the infrared channel of the two-photon microscope. A glomerulus is a strongly subcompartmentalized and inhomogeneous structure with segregated ON termination and dendritic zones (Kasowski et al., 1999; Kosaka and Kosaka, 2005). Moreover, there is no clear ultrastructural distinction between juxtaglomerular and intraglomerular neuropil (Pinching and Powell, 1971b,c) and the shape of a glomerulus often strongly deviates from a sphere. Therefore, we quantified the arborization patterns of cell types within subglomerular and juxtaglomerular shells, based on a “Sholl-type” analysis adapted to the individual glomerular surface. By this means we determined the relative dendritic innervation of glomeruli by the dopaminergic SAC type under debate as well as by classical PGCs.

## Materials and methods

### Animals and slice preparation

Rats and mice were decapitated under deep anesthesia with isoflurane according to the stipulations of the German law governing animal welfare (Tierschutzgesetz) and according to the EU directive 2010/63/EU, as approved by the Bavarian state government (Regierung von Oberbayern). Brains were removed and horizontal OB brain slices (300 μm thick) were prepared of juvenile wild-type (Wistar) and VGAT-Venus rats (VGAT: vesicular GABA transporter; line Venus-A, Uematsu et al., 2008) of either sex (postnatal days 11—18) as well as adult wild-type (WT) mice (postnatal weeks 16–40, BL-6) and adult DAT-GFP mice of either sex (postnatal weeks 16–24, DAT::CreER^T2^/CAG::GFP also in BL-6 background) (Nakamura et al., 2006; Rieker et al., 2011).

Both VGAT-Venus rats and DAT-GFP mice are based on mouse BAC transgenic lines (Uematsu et al., 2008). In the DAT-GFP transgenic line, GFP is expressed in a subset of dopamine transporter positive (DAT+) cells in adult animals after tamoxifen induction. The overlap between DAT and GFP has been established earlier (Ninkovic et al., 2010). Briefly, the DAT-GFP mice received intraperitoneal tamoxifen (T-5648, Sigma, St Louis, MO, USA) injections (15 mg/kg body weight; AZ 552-1-54-2531-144/07) on 5 consecutive days, the last one 10–20 days before slice preparation.

VGAT-Venus transgenic rats were generated by Drs. Y. Yanagawa, M. Hirabayashi and Y. Kawaguchi at the National Institute for Physiological Sciences, Okazaki, Japan, using pCS2-Venus provided by Dr. A. Miyawaki (Uematsu et al., 2008), RRID: RGD_2314361. In this rat line, fluorescent Venus protein is preferentially expressed in cells carrying VGAT, i.e., in GABAergic neurons: the localization of Venus-labeled cells across OB layers was found to be similar to that of GABA-positive cells; direct colocalization in the cortex yielded an overlap of 97% (Uematsu et al., 2008).

Brain slices animals were incubated in carbogen gas (95% O_2_, 5% CO_2_) infused artificial cerebrospinal fluid (ACSF, composition: 125 mM NaCl, 26 mM NaHCO_3_, 1.25 mM NaH_2_PO_4_, 20 mM glucose, 2.5 mM KCl, 1 mM MgCl_2_, and 2 mM CaCl_2_), in a heated water bath at 33°C for 30 min and then kept at room temperature (22°C) until experimentation.

To label dopaminergic cells in wild-type mice and rats, acute brain slices were incubated in ACSF containing 10 μM of the fluorescent pH-responsive probe FFN102 (Abcam, Cambridge, MA, USA) for 45 min (30 min at 33°C and a further 15 min at room temperature), adapted from the *in-vitro* protocol specified in Rodriguez et al. (2013). Before experimentation, FFN102-treated slices were washed with FFN-free ACSF (which was also used during electrophysiological recording and imaging) in the recording chamber for at least 15 min.

To specifically label glial cells along with FFN102 labeling in WT mice, WT rats and VGAT-Venus rats, acute brain slices were co-incubated in 10 μM FFN102 and 50 μM Sulforhodamine101 (Nimmerjahn et al., 2004; 45 min total incubation time, as described above). Before imaging, these slices were washed via perfusion of ACSF for at least 25 min.

### Two-photon imaging and electrophysiology

Fluorescence was recorded by two-photon laser scanning microscopy (TPLSM) on a Femto-2D microscope (Femtonics, Budapest, HU), equipped with a tunable, Verdi-pumped Ti:Sa laser (Chameleon Ultra I, Coherent, Glasgow, Scotland). The microscope was equipped with a 60x Nikon Fluor water-immersion objective (NA 1.0; Nikon Instruments, Melville, NY, USA), three detection channels (green fluorescence (epi and trans), red fluorescence (epi) and infrared light (trans)) and controlled by MES v4.5.613 software (Femtonics).

Fluorescent cells in rats (label Venus and/or FFN) and mice (label FFN or GFP) were identified in the green channel at an excitation wavelength of 730–760 nm (Venus, FFN) or 900 nm (GFP). Individual fluorescent cell bodies were patched in whole-cell mode with patch pipettes (resistance 6–8 MOhm), filled with an intracellular solution (composition: 130 mM K-methylsulfate, 10 mM HEPES, 4 mM MgCl_2_, 2.5 mM Na_2_ ATP, 0.4 mM NaGTP, 10 mM Na-phosphocreatine, 2 mM ascorbate). Electrophysiological recordings were made with an EPC-10 amplifier using Patchmaster software (both HEKA Elektronik, Lambrecht/Pfalz, Germany). For FFN102 and Venus experiments, the red fluorescent dye Alexa Fluor 594 (50 μM, Invitrogen, Carlsbad, CA, USA) was added to the intracellular solution to allow for the visualization of dendrites. In DAT-GFP+ cells, the calcium indicator OGB-1 (100 μM, Invitrogen) was added for both calcium imaging and neurite visualization. Fluorescence transients and image stacks were acquired at 800 nm laser excitation. Data were mostly collected from glomeruli within the medial surface of the OB.

All experiments were performed at room temperature (22°C). The patched JGCs were held in current clamp mode near their resting potential of −60 to −70 mV and the access resistance was monitored. Polarizing step pulses were applied for 500 ms each (first step −90 pA, increased by +30 pA for 10 steps, ending at +180 pA). In calcium imaging experiments, cells with a holding current value above 25 pA were rejected. A shift in baseline fluorescence F_0_ of more than 15% between the first and the last measurement of each region of interest (ROI) also led to a rejection of the experiment.

After sufficient filling of the dendritic tree (at least 15 min), stacks of scans of the entire cell were recorded at 1 μm z-resolution. Each scan included 2–3 images, recorded in the red (Alexa) and/or green (OGB-1, FFN) fluorescent channel and at the same time in the trans-infrared (IR) channel of the microscope, to gather information on both the dendritic tree and glomerular structure. The xy-resolution was 900 × 900 or 1000 × 1000 pixels with a pixel width of 0.098–0.24 μm, trading off between resolution and coverage of the stained cellular neurites and glomerular contours by the scanning window. All cells fit within one or two scanning windows and were fully sampled. In some instances we noted upon reconstruction that cells had been incompletely scanned, mostly because the stack's z-coordinate was not set deep enough. These neurons were not used for morphological analyses.

During calcium imaging experiments in DAT+ clasping cells, structures of interest were imaged in free line-scanning mode with a temporal resolution of ~ 1 ms. At a given dendritic location, several consecutive focal line-scans during somatically evoked single action potentials (sAPs) (by an injected current step of 1000 pA for 1 ms) or action potential trains (15 stimuli at 50 Hz) were recorded (duration 1.5 s), averaged and smoothed. Dendritic calcium transients ΔF/F were analyzed relative to the resting fluorescence F_0_, with their decay measured in terms of half-duration τ_1/2_ (Egger et al., 2003). τ_1/2_ values were capped at 5 s, because a higher value could not be reliably extrapolated. Post-hoc data analysis was performed using custom macros written in IGOR Pro 5 (Wavemetrics, Lake Oswego, OR, USA).

### Reconstruction and analysis of cellular morphology

To measure the cell body dimensions of juxtaglomerular neurons, we approximated the somatic shape as oval within single x-y-slices. We calculated the mean diameters d for any given soma from the slice with the largest oval cross-sectional area A and perimeter P as follows (cf. Heyt and Diaz, 1975):

$$\begin{array}{l} \text{d}=1.55{\text{A}}^{0.625}/{\text{P}}^{0.25} \end{array}$$

The detailed morphology of the dendrites of dopaminergic SACs and other glomerular cells was reconstructed with the Fiji plugin Simple Neurite Tracer (Longair et al., 2011) from the fluorescence z-stack scans. Thin, putative axonal structures could not be observed for all cells. If such putative axons were detected, they could not be followed for more than 100 μm, a drawback of reconstructions from live TPLSM data (Blackman et al., 2014; and results).

The following morphometric parameters were extracted from Simple Neurite Tracer analysis tools and other ImageJ calculations: 3D coordinates, total process length, soma dimensions, number of branches and end-points and average branch length. In this context, a branch is defined as a part of the cell bounded by any two junction points. Both spines and other endings are counted as terminal points. To prevent short protrusions from undersized cells to be classified as primary dendrites (cf. **Figure 4**), a primary dendrite was established according to the following criterion: It had to account for > 10% of the total process length and to stem from the cell soma or a main dendrite bifurcating no farther than 15 μm from the soma. All other morphometric analyses were derived or computed from those values, except for the arborization analysis (see below).

### Reconstruction of glomeruli for periglomerular and clasping cells

**Figure 1:**
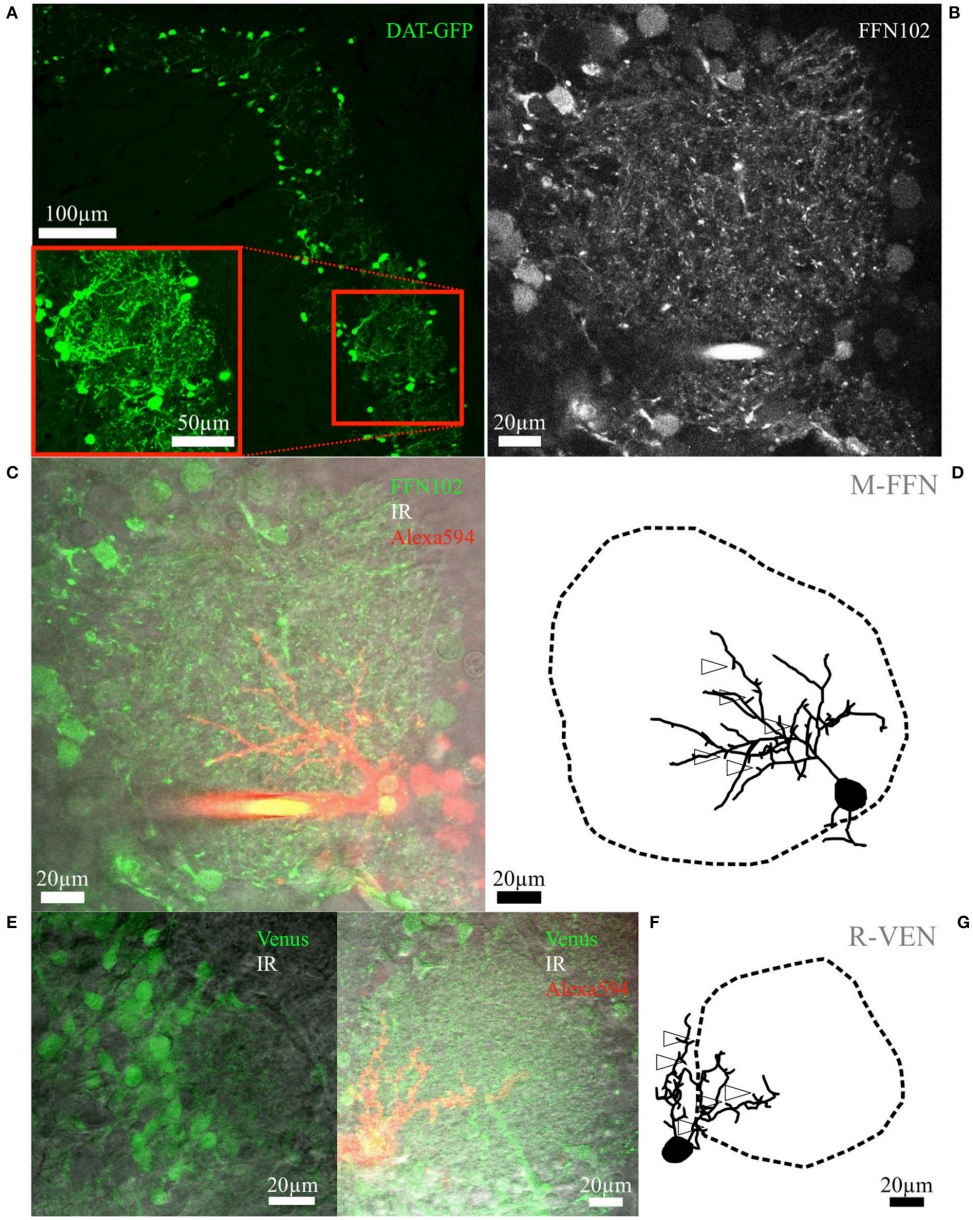
**Fluorescent cell labeling and reconstructions. (A)** Maximal z-projection scan of an OB slice from an adult mouse with tamoxifen-induced DAT-GFP+ cells surrounding the glomeruli. The inset shows a rescan at higher magnification. **(B)** Representative live FFN102 labeling of the glomerular and juxtaglomerular neuropil in an adult WT mouse brain slice. Green fluorescence channel of the TPLSM, with some brightly stained puncta within the glomerular neuropil as well as some labeled cell bodies at the glomerulus borders. **(C)** Same region in the trans-infrared detection (trans-IR) channel shown in gray overlaid with the green FFN102 fluorescence from **(B)** and the Alexa-594 stained, FFN102+ single neuron in red. **(D)** Maximal z-projection of the fully reconstructed cell from **(C)**, including an outline of the glomerulus. The open arrowheads indicate exemplary spines; not all spines are labeled for sake of clarity. The selected cell is the PGC also depicted in Figure 3B. Similar representations of cells and glomeruli are also used in Figures 2–4. **(E)** Representative VGAT-Venus labeling of the glomerular and juxtaglomerular neuropil in a juvenile rat in the green and the trans-IR channel. **(F)** Another VGAT-Venus example overlaid with the trans-IR channel shown in gray and the Alexa-594 stained, Venus+ single neuron in red. **(G)** The respective reconstruction of the clasping cell and its parent glomerulus from **(F)** which is also shown in Figure 2C. The open arrowheads indicate exemplary spines; not all spines are labeled for sake of clarity.

The glomerular contours were reconstructed from the TPLSM trans-IR image stacks with the ImageJ plugin TrakEM2 (Cardona et al., 2012). An additional glomerular counterstain was not required due to the good visibility of glomerular contours in the trans-IR-TPLSM channel and thus both dendritic and glomerular data could be gathered in the very same coordinate system, allowing for a precise arborization analysis (see Video S1). For FFN-stained slices or slices from Venus rats, the overlay of the FFN/Venus staining with the trans-IR channel yielded additional contrast between cell bodies/juxtaglomerular neuropil and intraglomerular neuropil (Figures 1C,F). Contours were determined by tracing the border between the ring-like arrangement of JGC somata and the intraglomerular neuropil border on the glomerulus inside for each successive z-scan (1 μm distance). The TrakEM2 “interpolate gaps” tool allows to interpolate the areas between two traced slices, which was sometimes applied for traced z-scans close to the midline of a glomerulus, yet not across more than 3 μm distance to ensure high structural accuracy. Even though the images became more blurry in deeper parts of the brain slice (depending on the brain slice quality, age of the animal etc.), the transition between contrast-rich somata and the grainy neuropil was still detectable for most scanned glomeruli. If the visibility of deep glomerular contours was compromised, the data was excluded from detailed analysis.

In 2 of the 30 cells sampled for arborization analysis the deeper part of the glomerulus was not scanned completely although the dendrites were fully recovered. In these cases the respective glomerulus was completed virtually in order to reduce errors in volume and average radius measurements (see below) by the following procedure: First the glomerular cross-sectional area at the deepest z-slice was determined and matched to the z-slice with the most similar area size of the top z-side of the glomerulus. Then the remaining scans on the top side until the end of the glomerulus were added to the incomplete part in mirrored order and aligned to the center of gravity of the deepest contour, resulting in an added volume of 10 and 28% of the total volume for the respective glomeruli.

### Arborization analysis and nomenclature of neurons

Prior to the methodical description of this novel approach, we define three auxiliary terms: (1) “glomerular neuropil” refers to the intraglomerular tissue, but not to the surrounding cell bodies or the juxtaglomerular neuropil, (2) a cell's “parent glomerulus” is either the only glomerulus contacted within its neuropil, or the glomerulus with the largest fraction of the cell's process length adjacent to its neuropil (within the juxtaglomerular'outer shell', see below). (3) The glomerular “intermediate zone” denotes the transition from the periglomerular cell body layer/juxtaglomerular neuropil to the intraglomerular neuropil; it has been characterized with light microscopy and at an ultrastructural level (Pinching and Powell, 1971b,c).

As to the nomenclature of neuronal subtypes, we have named the SAC subtype with dense dendritic arborizations clasping around other JGC somata “clasping cells” (CCs). The other prevalent cell type is the classical periglomerular cell (PGC) with dendritic arborizations in a single glomerulus. Further, small JGCs with an undersized dendritic tree are referred to as “undersized cells” (UCs). Detailed classification criteria are provided in the respective results parts (The clasping cell type, The periglomerular subtype, and Other small glomerular cell types).

For analysis of the glomerular arborization by a reconstructed dendritic tree, the reconstruction data were reformatted and then imported into IGOR via custom-written software. Pixel coordinates were converted into metric coordinates. Next, the coordinate systems of the two reconstructions—dendritic tuft and parent glomerulus—were aligned. After this step, 3-D voxel representations with a voxel side length of 1 μm were generated both of the dendritic tree (Hellwig, 2000; Egger et al., 2008) and of the parent glomerulus. The tuft voxel representation did not account for dendritic diameters.

In some instances there were gaps in the glomerular surface representation due to an insufficient density of data points in the reconstruction (mainly in contour lines that happened to run parallel to the x- or the y-axis for several μm). In these cases we used a custom-written filling algorithm which extended the reconstructed surface by 1–2 μm. The maximal error in glomerular volume due to this procedure was on the order of < 5%.

The voxel representations were used to determine the following parameters: (1) Total amount of 1 μm-voxels within a glomerulus, (2) glomerular volume, glomerular center of mass and mean glomerular radius, and (3) fractional arborization within shells as described below. For analyzing the relation between a glomerular layer neuron and its parent glomerulus, an intuitive approach would be to conduct a 3-D Sholl-type analysis (Sholl, 1953) originating from the center of mass of the glomerulus rather than from the soma of the neuron. However, since glomeruli were usually not spherical (or ellipsoid) in shape, we could not apply a straightforward 3-D analysis of arborization based on equally spaced spherical (or ellipsoidal) glomerular shells. Instead, shell volumes were calculated based on the real glomerular shape that is, in terms of expanding or shrinking the reconstructed glomerular surface by a certain radial distance from the center of mass of the glomerulus. The fraction of arborization was then determined by counting all tuft voxels of the 1 μm-voxel representation within the volume of a shell and normalizing the number to the total number of tuft voxels within the glomerular neuropil.

To allow for a quantitative analysis of intra- vs. juxtaglomerular arborization and for the inclusion of an intermediate zone we defined the following three volumes (cf. also **Figure 6A**): The “outer shell” was an extra layer with 15 μm thickness around the reconstructed glomerular surface, i.e., the border between glomerular neuropil and the juxtaglomerular cell bodies. The chosen shell thickness of 15 μm should encompass all somata in this juxtaglomerular volume, including the large dopaminergic lateral association subtype and ETCs (Nagayama et al., 2014). The glomerular neuropil was subdivided into (1) the “inner shell,” a volume extending for 15% of the glomerular mean radius from the neuropil-to-soma border toward the glomerulus centroid (choice of shell thickness see below) and (2) the “core,” i.e., the remaining inner volume. The combined inner and outer shell volume might thus account for the “intermediate zone,” while the combined core and inner shell volumes represent the intraglomerular neuropil of the parent glomerulus.

The value of the inner shell thickness was optimized by varying this parameter and analyzing the effect on the relative fraction of combined inner and outer shell innervation to the total innervation of all three volumes for clasping cells and PGCs and their standard deviations (see also **Figure 6C**). We found that the difference between CC and PGC fractions was the larger the thinner the inner shell was. However, the variance of both fractions across cells was most similar for a thickness of 15% of the glomerular mean radius, improving the statistics of the comparison. This value corresponds to 5.3 ± 1.2 μm (*n* = 30).

To better illustrate these data, we put them into the context of other well-known glomerular dendritic structures by including a data set from rat mitral cell apical dendritic tufts and their parent glomeruli. Mitral cells had been filled with Alexa Fluor 594 (50 μM; wild-type rats, P12–P16).

### Statistical analysis

Statistics were performed in SigmaPlot 13.0 (Systat Software, Inc., San Jose, CA, USA). Student's *t*-test or Mann-Whitney test was used for all comparisons between two groups except for paired data (e.g., within the same cell) where the Wilcoxon pair test was used. For comparisons of three groups, one-way ANOVA or Kruskal-Wallis-Tests were performed. For correlations, a linear regression analysis was utilized to determine *R*-values. Data are presented as mean values of parameters ± standard deviation (S.D.).

## Results

### Identification and reconstruction of dopaminergic and other small juxtaglomerular cells in rat and mouse olfactory bulb slices

To identify dopaminergic cells we used a tamoxifen-inducible GFP mouse line (DAT::CreER^T2^/CAG::GFP, cf. Rieker et al., 2011). GFP expression in adult mice showed labeled cells exclusively in the glomerular layer with strong fluorescence in cell bodies and upon high laser illumination also in dendritic processes (Figure 1A).

To gather dopaminergic JGCs also from rat and wild type mouse brain slices we attempted to use the acute label FFN102, which was shown to be a highly specific polar DAT and vesicular monoamine transporter 2 substrate in the ventral midbrain and striatal areas (Rodriguez et al., 2013). Thus, we expected FFN102 to specifically stain dopaminergic neurons in the OB glomerular layer, since there are no resident cells expressing monoamines apart from dopaminergic JGCs. However, in our hands FFN102 turned out to label the majority of JGCs, including subsets of periglomerular somata, and putative synaptic terminals (observable as bright puncta) throughout the glomerular layer of rat and mouse acute OB slices (Figures 1B,C). The labeled populations seemed to include mostly GABAergic neurons (colocalization with fluorescent Venus-labeled VGAT+ cells: 88 ± 6% overlap, *n* = 3 rats, see Figures 1E,F) and a few astrocytes (colocalization with Sulforhodamine101 (Nimmerjahn et al., 2004): 9 ± 2% overlap, *n* = 3 rats, *n* = 2 mice). A small subset of FFN+ cells were unlikely to be Venus+ or Sulforhodamine101+. Moreover, a considerable fraction of the FFN+ cells filled with fluorescent dye were classical PGCs (13 of a total 42 cells, see The periglomerular subtype (PGC) below, example cells in Figures 1C,D, 3B,D–F), whereas DAT+ cells never showed PGC morphology (*n* = 37 cells). Thus we conclude that FFN102 is most likely not dopamine-specific in the OB. Nevertheless, the FFN102 label simplified reconstruction of glomerular contours because of the broad staining of cell bodies (Figure 1B). Since FFN102 is not fixable, its overlap with immunohistological labels could not be tested, and therefore we could not prove directly that there is unspecific staining.

**Figure 2:**
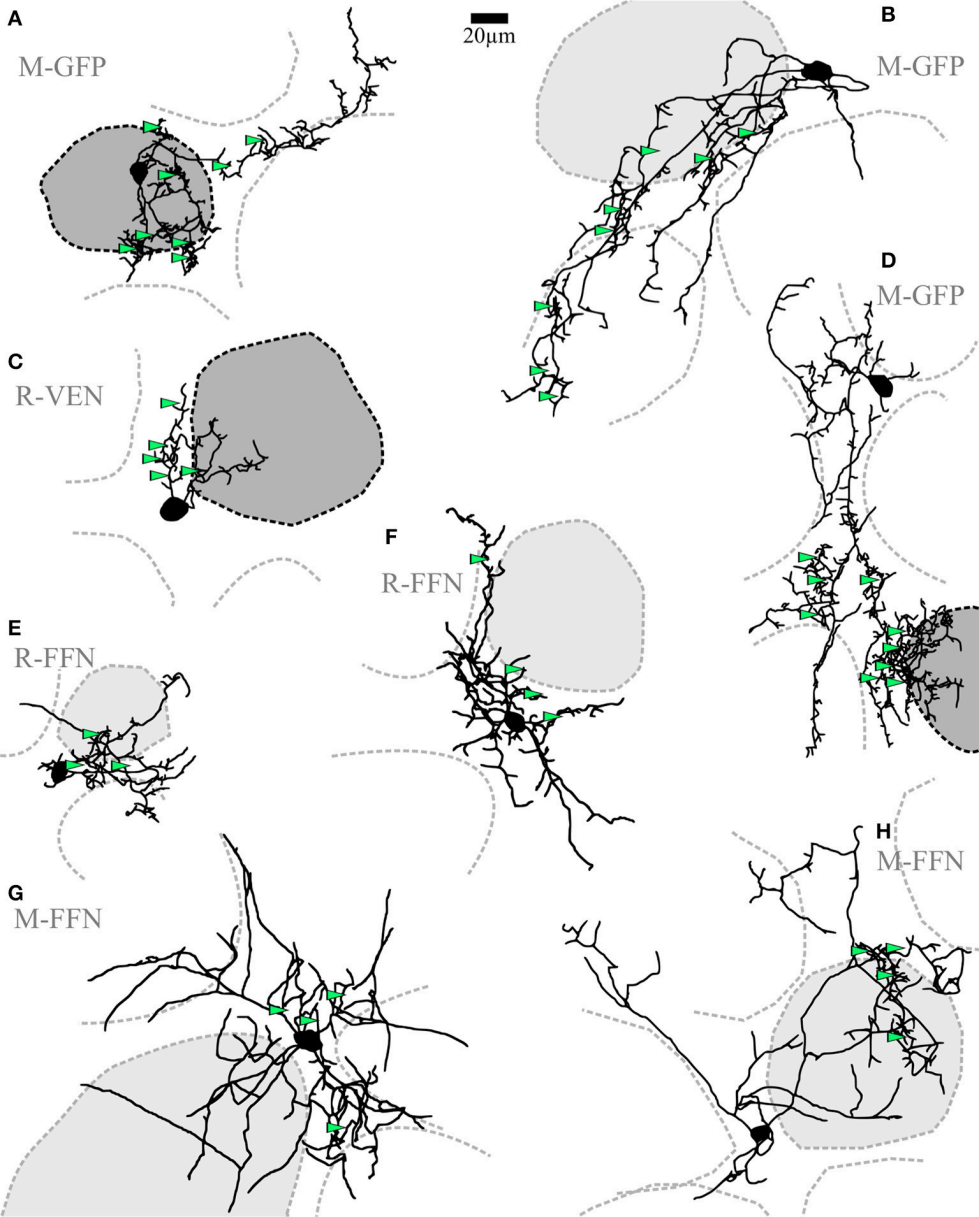
**Clasping cells (CCs)**. **(A–H)** Maximal z-projections of reconstructions of individual representative CCs with indicated surrounding glomeruli. The “parent glomerulus” is shaded for each cell (see methods). Darker shades indicate that the cell arborizes within the glomerular neuropil **(A,C,D)**, whereas light shades indicate non-innervated parent glomeruli. Non-shaded structures are surrounding glomeruli, which the dendrites do not ramify in and which do not constitute the parent glomerulus. Green arrowheads indicate the position of clasped JGC somata. Most dendritic structures including cell body clasps do not ramify in the intraglomerular neuropil, with the exception of a few superficially entering branches, also including dense clasp-like bundles **(A,C,D)**. R-VEN, VGAT-Venus+ cells in juvenile rats; R-FFN, FFN102+ cells in juvenile rats; M-FFN, FFN102+ cells in adult mice; M-GFP, DAT-GFP+ cells in adult mice.

**Figure 3:**
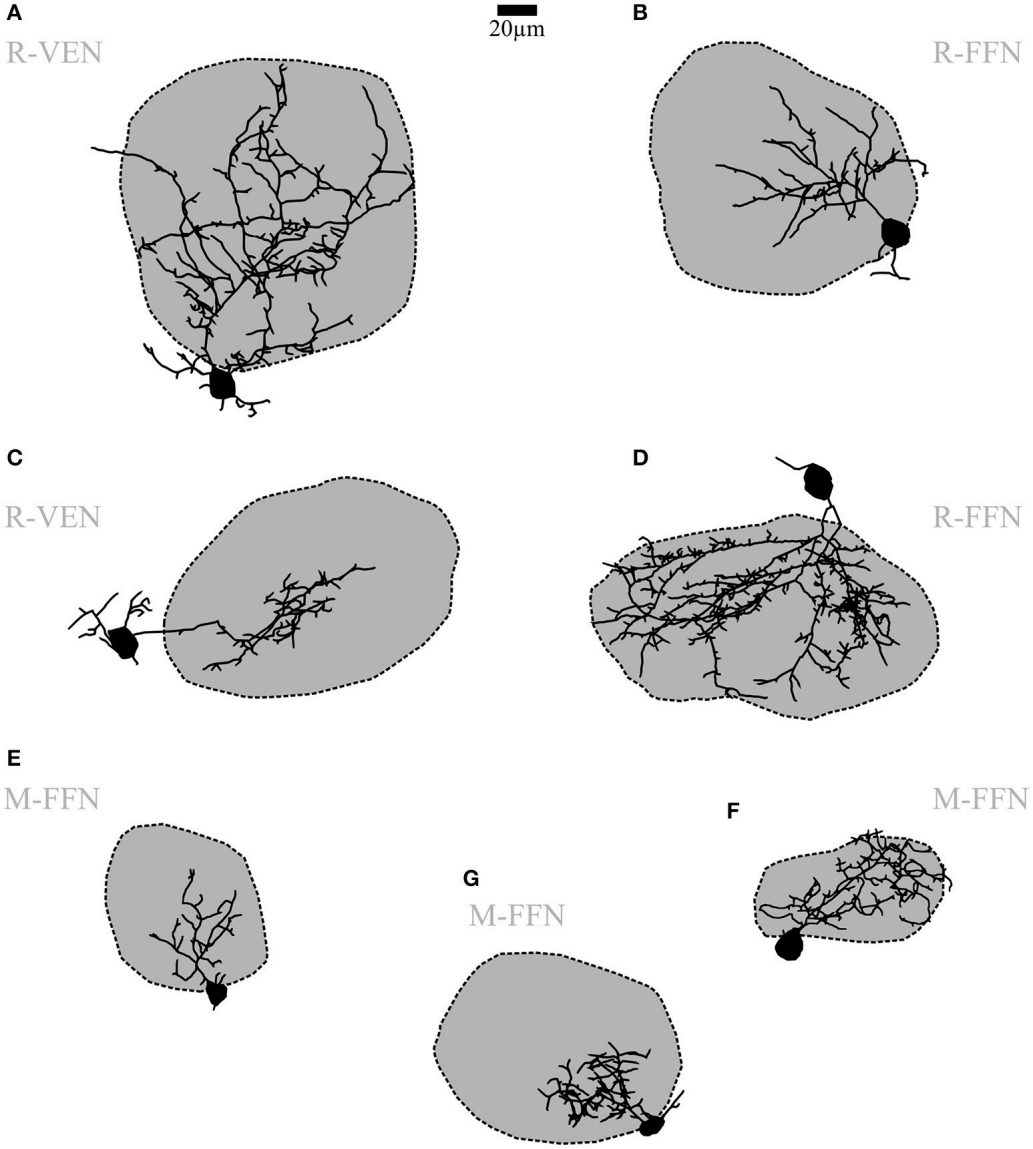
**Periglomerular cells (PGCs)**. **(A–G)** Maximal z-projections of reconstructions of individual representative PGCs with their corresponding contacted glomeruli. The dark shading indicates that dendrites contact the intraglomerular neuropil. Note the differing fractions of intraglomerular arborization with sparse or no juxtaglomerular processes, as well as the differing degree of branching and spine density across individual cells. R-VEN, VGAT-Venus+ cells in juvenile rats; R-FFN, FFN102+ cells in juvenile rats; M-FFN, FFN102+ cells in adult mice. Scale same size as in Figure 2.

**Figure 4:**
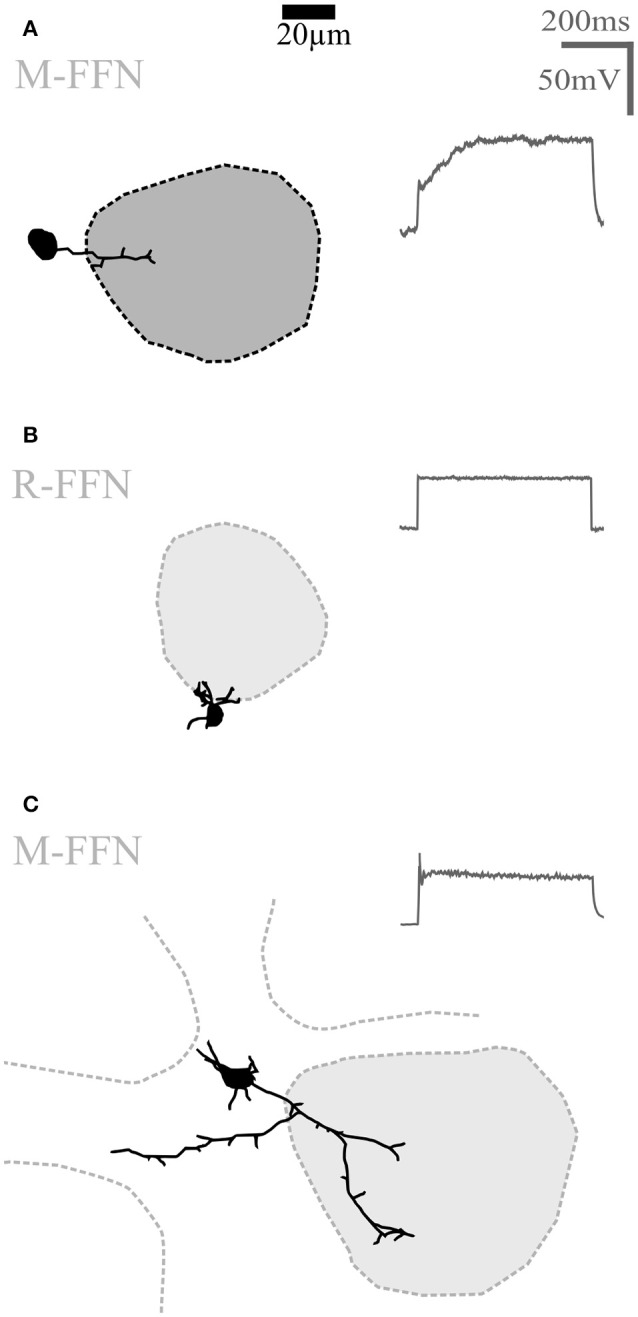
**Undersized cells (UCs)**. Maximal z-projections of reconstructions of representative UCs. Shading of glomeruli corresponding to Figures 2, 3. None of the reconstructed UCs showed truncations or signs of bad filling. UCs also never showed regular sodium action potential firing (voltage traces shown on the right in panels **A–C**) upon step depolarizations. **(A)** Morphologically very simple cell entering a single glomerulus. **(B)** Even smaller cell that does not enter the adjacent glomerulus. **(C)** Slightly larger cell compared to **(A,B)**, extending dendrites in the juxtaglomerular space. R-FFN, FFN102+ cells in juvenile rats; M-FFN, FFN102+ cells in adult mice.

For morphological reconstructions, DAT-GFP+ or randomly patched small glomerular cells (soma diameter 5–12 μm) in FFN102-stained slices or slices from VGAT-Venus rats were labeled intracellularly with fluorescent dyes via whole-cell patching and scanned using TPLSM. While the dendrites could be reconstructed, the fluorescence signal from thin, putative axonal structures was not bright enough except for the initial parts of some axons. In parallel, the surrounding tissue was scanned and the 3D-surface of the glomerular neuropil was reconstructed (see methods). This approach—simultaneous sampling of detailed individual cell structure and the surrounding glomerular tissue—allows to precisely determine neuronal morphometrics relative to glomerular contours (Figures 1D,G, 2–4, Video S1).

### Morphological features of small juxtaglomerular cells

#### The clasping cell type (CC)

Short-axon cells with densely ramifying dendritic specializations (clasps) were frequently observed among the GFP+ neurons in the DAT mouse (*n* = 19 of 37 patched cells). The primary CC dendrites (average number 3.5 ± 1.6, *n* = 16 CCs) were found to extend around several glomeruli for a few hundred micrometers in tortuous shapes. Most dendrites were found to be ramifying in the juxtaglomerular space of the glomerular layer (Figure 2). If these dendrites ever entered the glomerular neuropil (in 8 out of 16 fully reconstructed cells with parent glomerulus), they did so only in a restricted fashion, namely within mostly superficial volumes of one particular parent glomerulus. No substantial projection into any additional glomeruli was observed. Across cells there was no obvious orientation of the dendritic trees relative to the glomerular layer or the parent glomerulus, and the dendrites never entered the external plexiform layer. Taken together, the dendritic morphology of CCs closely resembles those of previously described small dopaminergic SACs of the glomerular layer (see discussion).

**Figure 5:**
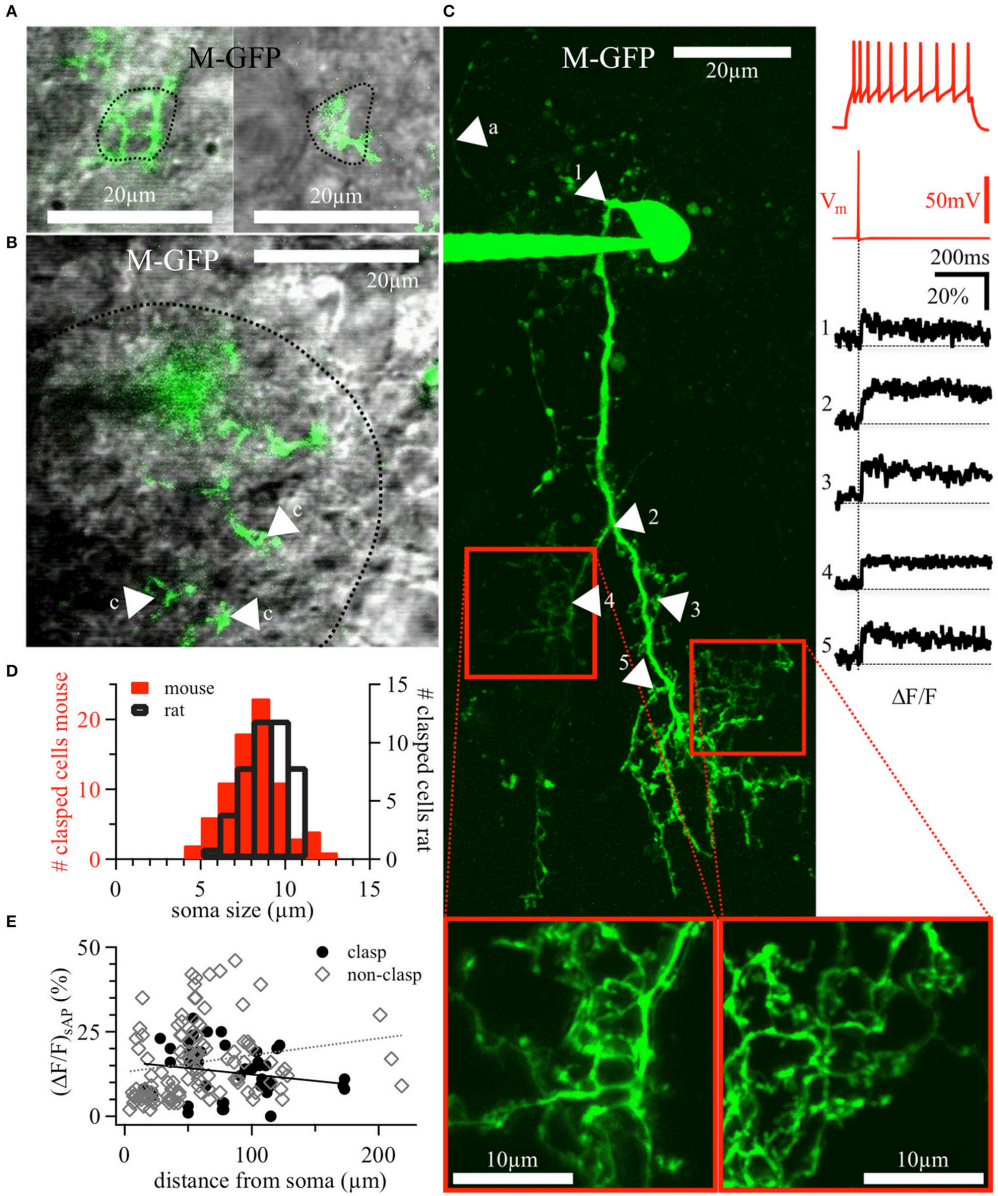
**DAT+ clasping cell dendrites: Clasp structures and AP-evoked Ca^**2+**^ signals. (A)** Two different clasping structures around cell bodies (left and right), with a dotted line indicating the somatic outline of the clasped cell—both structures are from a 20 weeks old DAT-GFP mouse (cell shown in panel **C**, the left clasping structure corresponds to the arrowhead number 4). **(B)** Exemplary, tight clasping structures (labeled “c”) within the glomerular neuropil (DAT-GFP mouse, 17 weeks old), with a dotted line indicating the glomerular border. **(A,B)** Show single z-plane scans from the green channel overlaid with the trans-IR channel in gray. **(C)** Maximal z-projection of the OGB-1 filled CC from Figure 2D (insets at bottom: magnified rescans of clasping structures). Numbered arrows in the scan correspond to the locations of the numbered averaged ΔF/F transients on the right, time-locked to the evoked somatic action potential (red trace directly above the black ΔF/F traces). The voltage recording on the top right shows this cell's accommodating firing pattern upon step depolarization (+90 pA current injection). In the scan, the arrow labeled with “a” points out a putative stained axon. It is thinner than the other, presumably dendritic, structures and bears no visible spines or clasps. **(D)** Distribution of the size of somata surrounded by clasps in juvenile rats (*n* = 45 clasped somata) and adult mice (both WT and DAT-GFP; *n* = 79 clasped somata). **(E)** Individual ΔF/F measurements versus the distance of their dendritic location from the soma (in *n* = 11 DAT-GFP+ mouse CCs; within clasps: black dots, *n* = 45 locations; within non-clasping dendrites: gray diamonds, *n* = 110 locations). No significant correlations emerged. M-GFP, DAT-GFP+ cells in adult mice.

The somatic clasps found on CC dendrites were identified according to the following criteria: (1) formed by at least two processes extending from the same parent dendrite, (2) embracing a single cell body from several spatial directions, such that (3) the soma is encompassed by the dendrite(s) for the length of at least half the mean cell diameter (see **Figure 5A**, Videos S2, S3). To classify as a CC in our study, a given cell had to show at least two of these clasp structures. Importantly, the CC morphology was also found in WT mice (*n* = 4, including 3 FFN+ cells) and rats (*n* = 5, all FFN+ cells) and in Venus+ cells from Venus rats (*n* = 7; see Figure 2 for examples).

Our approach did not allow to determine whether all CCs bear an axon (but see e.g., **Figure 5C**, methods and discussion). In any case, the clasp-bearing processes, which included most or sometimes all of the major neurites, are most likely dendrites since they always showed spine-like protrusions (see examples in **Figures 5C**, 1G). With exception of the clasp structures themselves, the neurites also otherwise resembled classical dendritic structures with respect to thickness, tapering and branching pattern.

**Figure 6:**
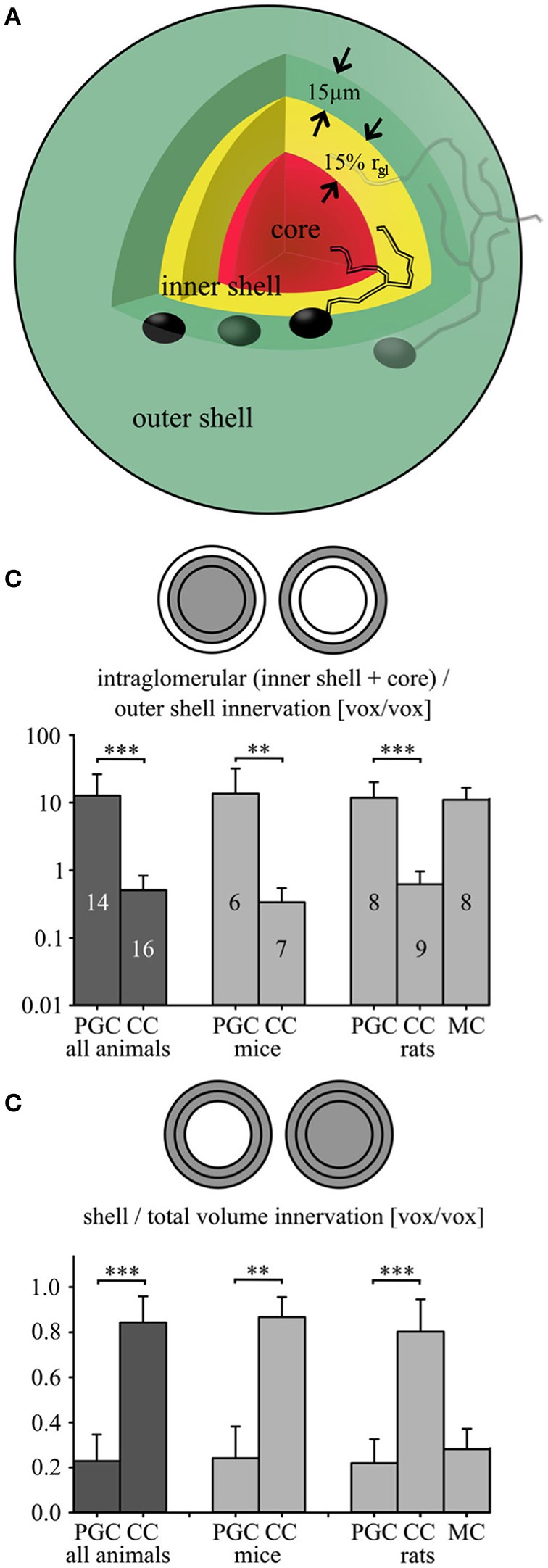
**Fractional glomerular arborization of clasping and periglomerular cells. (A)** Schema of the three analyzed parent glomerulus' volumes: an outer shell comprising directly adjacent juxtaglomerular cell bodies (thickness 15 μm; green), an inner shell (thickness 15% of the mean glomerular radius; yellow) and a core region (red; see methods). **(B)** Fractional arborization patterns of the PGCs and CCs [and additional rat mitral cells (MCs)]: Intraglomerular dendritic voxel number divided by the outer shell number for all pooled cells (left), or separated by species (middle and right). **(C)** Fractional arborization of the same cell types in the combined shell volume (inner + outer shell), relative to the total juxtaglomerular and glomerular volume (shells + core), again pooled (left) or separated by species (middle and right). ^**^P < 0.01; ^***^P < 0.005, Mann-Whitney-Test.

The soma size of CCs was in the medium range (average mean diameter 8.2 ± 1.7 μm for adult mice and 9.2 ± 1.8 μm for juvenile rats, *n* = 23 and *n* = 12, respectively). As mentioned above, clasping SACs were found in juvenile rats (WT and Venus+) as well as in adult mice (WT and DAT+) (**Figures 6A,B**).

#### The periglomerular subtype (PGC)

Within all cell populations except for DAT+ cells in the DAT-GFP mouse, we have regularly observed a uniglomerular subtype that corresponds to the previously described classical PGC morphotype (FFN+ cells in mouse: *n* = 2, FFN+ cells in rat: *n* = 11, Venus+ cells in Venus rat: *n* = 3; cf. Nagayama et al., 2014; Figure 3). Accordingly, we classified this subtype by the confinement of at least 50% of its total reconstructed neurite length to a single parent glomerulus (on average 88 ± 12%, *n* = 12 reconstructed cells; see methods). Within the glomerular neuropil these neurites had the appearance of classical dendrites and ramified extensively in roughly spherical volumes. Some cells arborized within almost the entire glomerular volume, while most only invaded a smaller part of the parent glomerulus, proximal to the cell body (7 vs. 5 of 12 cells, see e.g., Figures 3A,D,F vs. Figures 3B,C,E,G). These dendrites did not arborize in any other glomerulus apart from the parent glomerulus. The juxtaglomerular processes were thin and short, usually did not branch extensively nor show spines, and were not found to extend into other glomeruli's neuropil.

Most PGC dendrites bore spines or spine-like protrusions (75% of cells, see e.g., Figure 1D). Their density was variable across cells, with a tendency for a higher density on more extended cells. The soma size is in accordance with earlier descriptions of PGCs (average mean diameter 7.6 ± 1.3 μm for adult mice and 9.7 ± 2.1 μm for juvenile rats, *n* = 8 and 11, respectively) and did not significantly deviate from the soma size of CCs or UCs (for all further morphological comparisons, see Table 1). The number of primary dendrites did not differ between PGCs and CCs. Other parameters - number of branches and terminal points and overall branch complexity (total process length divided by number of branches)—showed no significant differences between CCs and PGCs.

**Table 1:** **Morphological parameters from cell reconstructions**.

**Parameter**	**Cell type**			
	**Animal**	**Clasping**	**Periglomerular**	**Undersized**
Soma mean diameter [μm]	mouse	7.6 ± 1.5	8.0 ± 1.3	7.1 ± 1.8
	rat	9.1 ± 1.9	9.2 ± 0.7	8.3 ± 1.4
	all	8.5 ± 1.9	8.6 ± 1.2	7.1 ± 1.7
Primary dendrites [#]	mouse	4.0 ± 1.5	2.7 ± 0.5	2.0 ± 1.2
	rat	3.1 ± 0.8	2.7 ± 1.2	2.9 ± 1.3
	all	3.5 ± 1.6	2.7 ± 0.9	2.6 ± 1.3
Total process length [μm]	mouse	2027 ± 620	763 ± 260	226 ± 163
	rat	1344 ± 580	1352 ± 716	351 ± 233
	all	1618 ± 670	1058 ± 598	310 ± 217
Branches [#]	mouse	275 ± 214	116 ± 54	34 ± 28
	rat	181 ± 144	246 ± 200	44 ± 33
	all	218 ± 175	181 ± 155	41 ± 31
End points [#]	mouse	132 ± 100	59 ± 28	18 ± 13
	rat	85 ± 66	120 ± 103	23 ± 16
	all	103 ± 81	90 ± 79	21 ± 15
Cell clasps [#]	mouse	6.3 ± 2.4	0	0.1 ± 0.3
	rat	5.9 ± 2.0	0	0.1 ± 0.2
	all	6.1 ± 2.1	0	0.0 ± 0.3
Average branch length [μm]	mouse	7.4 ± 3.7	6.8 ± 1.6	9.7 ± 8.3
	rat	7.8 ± 2.6	6.2 ± 1.5	8.4 ± 3.2
	all	7.6 ± 3.0	6.5 ± 1.5	8.8 ± 5.3
Cell number	mouse	6	6	10
	rat	9	6	22
	all	15	12	32

*Parameter numbers are stated as average (± S.D.). The mouse data include cells from both WT and DAT-GFP animals. The rat data include cells from both WT and Venus animals. The mouse data are from adult animals and the rat data from juvenile animals*.

#### Other small glomerular cell types

Here we describe in brief the remainder of the small soma cell types (< 11 μm in rat, < 10 μm in mouse) which we pooled as undersized cells (UCs; Figure 4) since this substantial population exhibited reduced dendritic complexity compared to the clasping SACs and the PGCs described above. These cells were observed within all populations (DAT-GFP+, FFN+, Venus+). On average, these cells bore rather short neurites (total process length on average 226 ± 163 μm for adult mice and 351 ± 233 μm for juvenile rats, *n* = 10 and 21, respectively; significantly smaller than CCs and PGCs, *P* < 0.001 each, Kruskal-Wallis-Test). UCs never showed regular spiking (never more than a single spike) upon step depolarization but rather strong inactivation (Figure 4). A few of these cells were more elaborate (total branch length > 500 μm for *n* = 6 of 31 cells), but still did not meet the criteria of either at least two clasps or an intraglomerular dendritic fraction of > 50%. The morphology of these cells was not analyzed in further detail.

### Structure and target cells of clasps

Clasping dendritic specializations around somata were highly abundant in juvenile rat (WT and Venus+) and adult mouse (WT and DAT-GFP+) CCs (6.3 ± 2.4 clasps per cell, *n* = 6 cells, and 5.9 ± 2.0, *n* = 9, respectively). In some instances dendritic structures including dense clasp-like structures entered the intraglomerular neuropil of a single parent glomerulus, mostly in superficial glomerular volumes (see e.g., Figures 2A,C,D and Figure 5B).

For any given CC, the cell bodies embraced by the clasps belonged to the juxtaglomerular zones of several adjacent glomeruli, always including the cell's parent glomerulus. The mean diameter of clasped cells (8.0 ± 1.6 μm, range 4.9–12.5 μm for adult mice, *n* = 78, and 8.8 ± 1.3 μm, range 5.8–11.0 μm for juvenile rats, *n* = 44, Figure 5D) overlaps with the diameter distributions of either other PGCs, SACs or ETCs (e.g., Nagayama et al., 2014). In DAT-GFP mice, the clasped cells were never GFP+ (*n* = 72); interestingly, in slices from WT rats and mice stained with FFN, clasped cells were also almost never FFN+ (*n* = 3 of 25 cells in 1 of 7 animals). In VGAT-Venus rats, the clasps contacted mainly non-fluorescent somata, except for a small fraction of Venus+ and thus most likely GABAergic cells (6 of 34 clasped cells in 3 of 10 animals).

### Excitability and dendritic Ca^2+^ dynamics of clasping cells

Dopaminergic JGCs are likely to feature active dendrites in order to sustain dendritic neurotransmitter release (Maher and Westbrook, 2008). To explore CC excitability, we first characterized the firing patterns of DAT-GFP+ CCs, which were almost exclusively of the accommodating spiking type (19 of 21 cells, see also example in Figure 5C; nomenclature according to McQuiston and Katz (2001), while the remaining two cells were firing no more than a single AP. All cells with accommodating firing pattern showed a low spiking threshold, with spikes observed already at the first depolarizing current injection step of 30 pA in 15 of 19 neurons and at 60 pA in all of the 19. Rat CCs responded in a similar manner (*n* = 3 cells, all accommodating spiking at low thresholds). We never observed bursting firing patterns, that were previously found to be characteristic for ETCs and also for a subset of classical PGCs, but less so for SACs (McQuiston and Katz, 2001; Hayar et al., 2004a).

The dendritic excitability and Ca^2+^ dynamics of CCs was tested by two-photon imaging of dendritic Ca^2+^ entry mediated by backpropagating APs in DAT-GFP+ cells filled with the Ca^2+^-sensitive dye OGB-1 (100 μM). Single somatically evoked APs (sAP) elicited substantial calcium influx throughout most of DAT-GFP+ CC dendrites (Figure 5C right). These (ΔF/F)_sAP_ signals were not saturating, since trains of 20 APs at 50 Hz recorded in the same locations resulted in larger ΔF/F transients, which usually reached a plateau during the train stimulation at a level expected for 100 μM OGB-1 (sAP 15 ± 10% ΔF/F vs. train 150 ± 74% ΔF/F in 155 locations in 11 CCs, *P* < 0.001, paired *t*-test). In general, ΔF/F transients decayed with a very slow half–duration τ_1/2_ = 2.5 ± 1.2 s (*n* = 94 locations in 11 CCs).

There was no evidence for strong attenuation of backpropagating APs since (ΔF/F)_sAP_ did not decrease with distance from the soma; rather, individual (ΔF/F)_sAP_ amplitudes were not correlated with the distance of the recording site from the soma for clasps, non-clasping dendrites or all data pooled (n.s.; Figure 5E).

In CCs that contacted intraglomerular neuropil, calcium signals measured in dendrites within glomeruli were not significantly different from those outside [data from above split into the two groups: glomerular mean (ΔF/F)_sAP_ = 15 ± 5%, *n* = 18; extraglomerular mean (ΔF/F)_sAP_ = 16 ± 13%, *n* = 141; Mann-Whitney test, n.s.], which was also true for the decay half-durations. Neither were (ΔF/F)_sAP_ amplitudes in clasps different from those in non-clasping dendrites across all CCs [data from above split into the two corresponding groups: clasp mean (ΔF/F)_sAP_ = 13 ± 8%, *n* = 45; non-clasp mean (ΔF/F)_sAP_ = 16 ± 11%, *n* = 111; n.s.]. Decay half-durations were also not significantly different in clasping vs. non-clasping structures (data not shown). Finally, there was also no significant trend for (ΔF/F)_sAP_ and τ_1/2_ in paired data sets (clasps versus non-clasping dendritic locations averaged within the same cells, *n* = 6 cells, Wilcoxon test, n.s.). We observed no correlations of any of the parameter combinations of ΔF/F, distance or decay half-duration in either of the populations (not shown).

In summary, these observations indicate a robust, slowly decaying AP-mediated Ca^2+^ entry into both conventional dendritic and clasp subcompartments of CCs.

### Analysis of dendritic arborization relative to glomeruli for clasping and periglomerular cells

CCs and PGCs in both juvenile rats and adult mice were similar in terms of several common morphological parameters—soma size, number of primary dendrites, total process length (only in rats), number of branches and terminal points or overall complexity (see Table 1). Therefore, we sought to discriminate these subtypes via the differential arborization pattern within the intra- and juxtaglomerular space, in particular in relation to their respective parent glomerulus. To quantify the arborization pattern we performed a 3-D-overlay of neuronal reconstructions with the reconstruction of their parent glomerulus and determined the relative dendrite distribution in three selected volumes of the glomerulus: core, inner and outer shell (Figure 6A; see methods).

First, the fraction of the dendritic voxels of the glomerular neuropil (inner shell + core) to the tuft voxels within the outer, juxtaglomerular shell (Figure 6B, Table 2, see also methods), was significantly lower in CCs than in PGCs (*P* < 0.001; Mann-Whitney test for all comparisons in this paragraph). Similar results emerged for the individually investigated species groups (both adult mice and juvenile rats: *P* < 0.005). Second, to account for the “intermediate zone” and for possible errors in determining the glomerular neuropil border we also counted all voxels within the entire shell region (inner + outer shell), and divided them by the total number of voxels within the complete volume of all three (core + inner + outer shell). This fractional arborization also displayed significantly less voxels for CCs compared to PGCs (*P* < 0.001, Figure 6C, Table 2). Again, the individual investigated species groups yielded similar results (adult mice *P* < 0.005; juvenile rats *P* < 0.001). Briefly, both measures of glomerular arborization proved to be very distinct for the cell types, i.e., CCs contact almost exclusively the shell regions, whereas PGCs primarily contact the inner shell and the core.

**Table 2:** **Morphological parameters from fractional innervation analysis**.

**Parameter: Fractional innervation (Figure 6A)**	**Cell type**			
	**Animal**	**Clasping**	**Periglomerular**	**Mitral**
Glomerular vs. outer shell	mouse	0.32 ± 0.20	13.6 ± 18.3	
	rat	0.62 ± 0.34	12.1 ± 8.9	11.0 ± 5.6
	all	0.49 ± 0.32	12.7 ± 13.1	
Core vs. complete shell	mouse	0.87 ± 0.09	0.24 ± 0.14	
	rat	0.80 ± 0.14	0.22 ± 0.11	0.28 ± 0.09
	all	0.83 ± 0.12	0.23 ± 0.12	
Cell number	mouse	7	6	
	rat	9	8	8
	all	16	14	

*Parameter numbers are stated as average (± S.D.). The mouse data include cells from both WT and DAT-GFP animals. The rat data include cells from both WT and Venus animals. The mouse data are from adult animals and the rat data from juvenile animals. For the definition of the parameters see Figure 6A*.

To further illustrate the outcome of the fractional innervation analysis, we included a set of juvenile rat mitral cell glomerular tuft data (*n* = 8). For both fractions, the mitral cell tuft arborization pattern results closely matched those for PGCs (no significant differences) and were significantly different from those for CCs (core + inner shell vs. outer shell *P* < 0.005, both shells vs. total volume *P* < 0.001, cf. Figures 6B,C).

## Discussion

Within our sample of small JGCs, we have consistently observed three morphological subtypes across various mouse and rat strains: (I) Clasping cells, identified first as DAT+ cells in a transgenic mouse line; they resemble the classic small dopaminergic SAC subtype that has been reported previously based on TH staining. All of these short-axon-like cells showed dendritic structural specializations that constitute somatic clasps, thus the term clasping cell (CC). (II) Classic periglomerular cells (PGCs), which in our sample always showed a uniglomerular innervation. (III) Undersized cells with respect to their dendritic morphology and reduced excitability (UCs).

### Reconstruction of dendrites and glomeruli for arborization analysis

In comparison to the classical biocytin-based reconstruction methods, detailed reconstructions based on fluorescence measurements in acute slices with TPLSM do not allow for complete recovery of very fine processes such as axons. However, they are superior to the classical biocytin-based methods with respect to efficiency and to z-shrinkage, since processes are reconstructed from images of living tissue. Parameters such as volumes, dendritic lengths and arborization densities can be accurately determined (Egger et al., 2008; Blackman et al., 2014). Also, reconstruction of glomerular contours from embedded material requires a counterstain, whereas in the acute slice preparation glomerular borders can be detected without extra staining.

Previously, the glomerular arborizations of projection neurons in the silkmoth were characterized in a similar manner (Kazawa et al., 2009; Namiki and Kanzaki, 2011). In extension of this approach, we have performed the innervation analysis in relation to different glomerular subvolumes (shells vs. core, Figure 6), enabling us to quantitatively compare the specific dendritic arborization patterns of CCs, PGCs and mitral cell apical tufts.

CC dendrites were found to almost exclusively ramify in the juxtaglomerular space, with a subset of cells (approximately 50%) projecting in addition into a single parent glomerulus. This glomerular projection consisted of a minor fraction of the CCs' total dendritic length and was mostly restricted to the glomerulus' superficial volume. We hypothesize that this superficial projection might correspond to a targeting of the so-called “intermediate zone,” which denotes the transition between juxtaglomerular and intraglomerular neuropil as described by Pinching and Powell (1971b,c).

Due to this mostly juxtaglomerular dendritic arborization, most sensory synaptic activation of CCs is likely to be provided in feed-forward mode via ETCs, which was also recently corroborated for dopaminergic JGCs in general (Kiyokage et al., 2010; Adam et al., 2014; Banerjee et al., 2015). A direct ON activation of CCs could thus occur only for the CCs with arborization within their parent glomerulus, provided that ON fibers indeed innervate the glomerular shell region that constitutes the major target of CC dendrite arborization according to our analysis and that might correspond to the “intermediate zone” (Pinching and Powell, 1971b,c).

In contrast to CCs, the dendrites of PGCs almost exclusively arborized inside the intraglomerular neuropil. Thus, these neurons are expected to receive inputs from intraglomerular synapses. Synaptic interactions with several cell types include symmetrical synapses from and onto glomerular interneurons, as well as dendrodendritic (sometimes reciprocal) arrangements with excitatory MTCs and presynaptic inhibition of olfactory sensory neurons (Chao et al., 1997; Kasowski et al., 1999; Kosaka and Kosaka, 2005). It remains to be elucidated if the partial and global glomerular innervation patterns observed here reflect also functional differences.

The arborization analysis approach taken here could also easily be generalized to investigate the arborization of glomerular layer cells in further glomerular subcompartments such as the segregated termination zones of the ON. It has been shown that some PGC subtypes arborize specifically within these termination zones (type 1 cells, Kosaka and Kosaka, 2005), whereas other subtypes only contact areas of the glomerular neuropil not contacted by the ON (type 2). To establish the specific overlap with or proximity to ON termination zones, labeled PGC dendrites could be analyzed in relation to ON terminals that were stained e.g., by nasal fluorescent labeling (Wachowiak and Cohen, 2001).

### Classification of cell types

Concerning clasping SACs, Kiyokage et al. (2010) described two dopaminergic subtypes based on their arborization patterns, termed oligoglomerular, and polyglomerular, which constitute the majority of what is subsumed under the term “glomerular SACs” in the recent literature (cf. also Nagayama et al., 2014). Whereas Kiyokage et al. (2010) report an arborization of neurites (including both axons and dendrites) within several or many glomeruli for both the larger polyglomerular, and the smaller oligoglomerular subtype, we found the dendrites of clasping SACs to contact no more than the intraglomerular neuropil of a single glomerulus, if at all.

This apparent discrepancy might be explained by the following technical differences: (1) Their label for dopaminergic cells was TH, versus DAT in our study—two markers which so far have never shown perfect overlap in the OB (Cave and Baker, 2009; Banerjee et al., 2015). (2) Kiyokage et al. (2010) reconstructed detailed morphologies in biocytin-filled cells in fixed slices with a confocal microscope, whereas we recorded image stacks in acute slices with TPLSM. Since the axons of short axon-like cells are particularly thin (hence also the historical insufficient axonal staining and misnaming of SACs), we presume that the fluorescence from these structures was mostly too weak for sufficient resolution in our scans, (see also Blackman et al., 2014), since we observed thin, axon-like processes without spines in only a subset of cells and could not follow them for more than approximately 100 μm. On the other hand, the biocytin method is much more sensitive and therefore should recover axonal processes (also pointed out by Kosaka and Kosaka, 2011), although Kiyokage et al. (2010) chose to not differentiate between dendrites and axons because of their similar appearance. Notably, so far the existence of axons has not been demonstrated directly for dopaminergic juxtaglomerular neurons except for the large lateral association neurons (Chand et al., 2015). (3) Finally, whether a glomerulus was classified as contacted or not might also have differed, due to the different reconstruction technique and level of resolution of glomerular contours. Moreover, the distinction of juxtaglomerular and intraglomerular neuropil is difficult *per se* because of the interdigitating “intermediate zone” (Pinching and Powell, 1971b,c).

Nevertheless, oligoglomerular cells and CCs might belong to the same population for several reasons. First, although Kiyokage et al. (2010) have not described clasp-like structures, some of their cells appear to bear clasps (e.g., their Figure 5B, top panels). Since clasping dendrites—untypical for conventional dendrites—also may “turn backwards” toward the soma, the clasps might also have contributed to the difficulty in differentiating axons and dendrites in their study. Second, the extended oligoglomerular innervation might be performed by the CC's axon (which is lacking from our reconstructions), while the dendrites project mostly into the juxtaglomerular space as described above. In line with this view, DAT+ cell bodies were reported to respond to odors in a sparse fashion which was highly correlated across a given glomerulus (Banerjee et al., 2015), and the glomerular layer odor response maps of GCaMP-labeled TH+ cells were rather focal and did not broaden with increasing odor concentrations, in contrast to signals from MTCs (Wachowiak et al., 2013)—observations that are most parsimoniously explained as the result of a predominantly local dendritic innervation. Thus, we would like to propose that CCs and “oligoglomerular DAergic JGCs” represent (or at least are subgroups of) the very same subtype of dopaminergic SACs. To ultimately verify this hypothesis a follow-up study would be required in which CCs are filled with biocytin and co-stained with an axonal or dendritic marker, such as immunohistochemical labeling of the axon initial segment-specific protein βIV-spectrin (Kosaka and Kosaka, 2011).

To identify DAT+ cells in WT rat and mouse brain slices, we turned to a novel *in-vitro* marker, FFN102, that had shown a very high specificity as established by the overlap with DAT+ immunoreactive cells in the striatum and ventral midbrain (Rodriguez et al., 2013). However, this specificity could not be reproduced in OB brain slices of mice and rats. Small JGCs stained by FFN102 included predominantly SACs and undersized JGCs, but also a substantial fraction of classical PGCs, which are not among the dopaminergic JGCs according to Kiyokage et al. (2010), and also were not observed in our sample of DAT-GFP+ cells. Testing the compound in VGAT-Venus+ transgenic rats revealed an overlap with most Venus+ cells (~ 90%), and both FFN102 and Venus labeling were observed in the majority of JGCs (cf. Figure 1), whereas several groups have reported that the fraction of dopaminergic neurons of the total JGC population is on the order of 10–30%, depending on the age of the animal (Ninkovic et al., 2007; Panzanelli et al., 2007; Parrish-Aungst et al., 2007; Adam and Mizrahi, 2011). Therefore, in our hands FFN102 is most likely not specific for dopaminergic cells in the OB; the uptake mechanism of this dye into bulbar cells remains to be elucidated.

The undersized cells (UCs) observed within all animal groups are unlikely to be insufficiently filled cells because they usually filled quickly and showed no truncations. Similarly undifferentiated cells have already been observed in the OB and classified as immature neurons based on morphological and physiological analyses (granule cells: Carleton et al., 2003; PGC/JGCs Mizrahi, 2007; Kovalchuk et al., 2015). These findings are related to the phenomenon of massive adult neurogenesis of certain local interneuron subtypes in the rodent OB, including granule cells, PGCs and dopaminergic and other types of SACs (Lledo et al., 2006; Ninkovic et al., 2007).

The UCs in our sample did either not fire proper Na^+^ spikes or at best one, in line with observations in young neurons in the aforementioned studies. Therefore, UCs might correspond to immature adult-born juxtaglomerular interneurons, including DAT+ cells, which are not yet completely integrated into the glomerular circuitry. Alternatively, UCs might constitute pre-apoptotic cells, since reportedly a large number of new-born arriving cells gets eliminated rather than integrated into the bulbar circuitry (Winner et al., 2002). In any case, we would like to argue that the existence of this cell population reflects the participation of certain subtypes of dopaminergic and other juxtaglomerular neurons in ongoing adult neurogenesis and remodeling in the OB.

With respect to JGC subtype composition, we found all the described cell types—CCs, PGCs, and UCs—in both juvenile rats and adult mice. While the overall number of cells in this study is too small for a quantitative analysis of subtype composition across these two populations, there was a tendency for relatively more CCs and less UCs and PGCs in adult mice whereas juvenile rats showed equal numbers of CCs and PGCs and higher numbers of UCs. This observation might be explained by an increasing relative fraction of CCs with age due to neurogenesis, although interspecies differences could also play a role (Kosaka and Kosaka, 2007). Another reason for fewer UCs in mature mice could be a faster maturation of integrating cells within the glomerular layer. Age-related shifts in subtype composition have been previously observed for various bulbar interneurons (Ninkovic et al., 2007; Batista-Brito et al., 2008; Adam and Mizrahi, 2011).

### Targets and function of clasp structures

Whether clasps indeed directly contact the embraced somata—as implied by their appearance—requires confirmation by ultrastructural analysis. Such an analysis would also elucidate the precise type of interaction—via dendrosomatic, somatodendritic or electrical synapses. Which cell types might be targeted by clasps? Since in the DAT-GFP mice all the clasped cells were GFP-negative, it is highly unlikely that dopaminergic cells themselves are targets of this specific structure. Previous studies have described functional influence of dopaminergic cells on MTCs (Bundschuh et al., 2012; Livneh et al., 2014; Banerjee et al., 2015), which can be excluded as candidate partners because of their soma size and location. In VGAT-Venus rats, the targets comprised only a small fraction of Venus+ cells. Thus, we speculate that the preferential clasp targets might be glutamatergic juxtaglomerular neurons, such as ETCs that are known to be postsynaptic to DAergic SACs (Liu et al., 2013), and glutamatergic SACs (Brill et al., 2009). Both cell types would also conform to the measured soma sizes of clasped cells (Figure 5D). Moreover, as of yet unidentified other glomerular cells and small glial cells could be among the targets of those dense dendritic structures.

As to the function of clasping cell dendrites, we hypothesized that these structures would be highly excitable. Highly active dendrites are rather common within the circuitry of the OB, due to its various dendrodendritic synaptic interactions. In several neuron types, like mitral cells and ETCs (Hayar et al., 2004a), Ca^2+^ dynamics evoked by (backpropagating) action potentials, (ΔF/F)_AP_, have been used as a readout for the presence of active dendritic conductances and thereby as an indication of dendritic transmitter release (Ludwig and Pittman, 2003). Other bulbar neurons include the GABAergic granule cells that show considerable (ΔF/F)_AP_ especially within their output region in the apical dendrite (Egger et al., 2003). However, it is not necessarily true that all OB neuron subtypes display effective backpropagation of single action potentials (Zhou et al., 2006).

Here we find that dendrites of DAT-GFP+ CCs also might fit the notion of releasing dendrites since they showed substantial (ΔF/F)_AP_, which did not decrease with distance from the soma. Moreover, the (ΔF/F)_AP_ signals decayed extremely slowly, which cannot be explained via buffering by the added dye and thus could promote asynchronous release of transmitter, similar to what has been suggested for granule cells (Egger and Stroh, 2009). Whether this slow decay is a result of slow extrusion or a very high buffering capacity remains to be elucidated. Dopaminergic JGCs have been shown to independently release GABA and dopamine from their dendrites (Borisovska et al., 2013; Brill et al., 2016). If dopamine gets released only upon sufficiently strong or prolonged stimulation (Maher and Westbrook, 2008; Bundschuh et al., 2012), it is interesting to note that the remarkably slow decay of Ca^2+^ reported here will result in strong summation of both postsynaptic and AP-evoked Ca^2+^ transients within a time frame of seconds. Hence, a sequence of action potentials, whether combined with local glutamatergic input or not, might foster release of dopamine, even if it occurs at a low frequency such as e.g., the prominent bulbar theta rhythm (Hayar et al., 2004b).

If clasps were indeed output structures, they would provide another example for chemical dendrosomatic synapses, which so far in the bulb have been described between VIP+ interneurons and MTC somata (Crespo et al., 2002) and also between type S granule cell dendrites and mitral cell somata (Naritsuka et al., 2009). Provided that the targeted cells are indeed neurons with axons, dendrosomatic clasps could exert strong influence on their output due to their close vicinity to the axon hillock. However, we did not observe any significant differences in (ΔF/F)_AP_ signals in clasping versus non-clasping dendritic compartments. Thus, it seems unlikely that clasps could be preferred release sites compared to the remainder of the dendrite.

Instead, or on top of being output structures, somatic clasps might also sense molecular signals from resident structures. Migrating cells rely on many chemical signals for orientation and survival on the way to full functionality (Whitman and Greer, 2007; Kovalchuk et al., 2015). Therefore clasps might serve to guide recently born CCs to their final spatial position in the glomerular layer. In this function, clasps might even be transient in nature, helping the cell to survive, migrate or integrate into the glomerular circuitry. Such a transient function could be related to the reported increased sensitivity and odorant promiscuity of integrating adult-born cells in the OB (Livneh et al., 2014).

## Ethics statement

Tamoxifen-induction experiments were approved by the Regierung von Oberbayern (AZ 552-1-54-2531-144/07). All other procedures did not require approval according to German animal welfare laws.

## Author contributions

All authors take responsibility for the integrity of the data and the accuracy of the data analysis. Study concept and design: WB, VE. Acquisition of data: WB, TO, and ML, histology: WB and JN. Analysis and interpretation of data: WB, VE, TO, and ML. Figure preparation WB (supplement also TO). Drafting of the manuscript: WB and VE. Obtained funding: VE, JN. Administrative, technical, and material support: WB, TO, VE, ML, JN. Study supervision: WB and VE.

## Funding

This work was funded by the DFG-SPP 1392 (Integrative Analysis of Olfaction; VE, JN) with additional equipment funding by LMU-GSN (Graduate School of Systemic Neurosciences) and DFG-SFB 870.

### Conflict of interest statement

The authors declare that the research was conducted in the absence of any commercial or financial relationships that could be construed as a potential conflict of interest.

## Supplementary material

Video S1 **3-D visualization of a reconstructed periglomerular cell and its contacted reconstructed glomerulus**. The depicted cell in this video corresponds to the cell shown in Figure 3D.

Videos S2, S3 **Visualization of clasping structures in z-stack movies**. Videos depict successive focal z-scans in TPLSM stacks. Each of the videos shows dendritic structures of a patched cell “clasping” around a targeted cell body. The red PMT image shows the dendritic structure, the trans-IR image (light-blue with dark contrast) shows the juxtaglomerular neuropil including cell bodies. **S2** shows a very complex clasping phenotype around a single cell body (in the center of the video). In this cell, the nucleus can be easily spotted. **S3** shows a less strongly clasped soma in the center of the video. The cell outline is well defined in this example.
